# Social Isolation and Well-Being Among Middle-Age and Older Adults: Before and During the COVID-19 Outbreak

**DOI:** 10.1093/geroni/igab046.141

**Published:** 2021-12-17

**Authors:** Lydia Li

## Abstract

This symposium brings together five studies that examined the relationship between social isolation and well-being. Two used pre-COVID data from the Health and Retirement Study (HRS). One aimed to identify patterns of social isolation trajectory in a 9-year period, where social isolation was conceptualized as a multidimensional construct. It identified four distinct patterns, and the pattern had a gradient relationship with health outcomes. Another examined the association between self-perceptions of aging (SPA) and social well-being among older adults. It found that positive SPA predicted increased social connectedness and reduced loneliness in four years. Two other studies were based on a longitudinal survey (COVID-19 Coping Survey) that began in April 2020. One reports that adults 55+ with comorbidity at pandemic onset had persistently elevated depressive symptoms in a 6-month period, regardless of their social isolation level. Another paper suggests that physical isolation at pandemic onset was associated with elevated symptoms of depression, anxiety, and loneliness throughout the following six months. The fifth paper was based on two-wave data—2019 survey and 2020 COVID supplement—from the National Aging and Health Trend Study (NAHTS). It found that older adults who were very socially isolated and completely homebound before the pandemic experienced less psychological distress during the outbreak than those who were very socially integrated and not homebound. The five studies highlight the multiple dimensions of social isolation, their antecedents and development over time, and their role in shaping mental health in a pandemic context.

